# Does injury risk increase when youth athletes start to study at a sports high school?

**DOI:** 10.1136/bmjsem-2023-001686

**Published:** 2023-11-03

**Authors:** Linda Ekenros, Cecilia Fridén, Philip von Rosen

**Affiliations:** 1 Department of Neurobiology, Care Sciences, and Society, Karolinska Institutet, Huddinge, Sweden; 2 Department of Women's and Children's Health, Karolinska Institutet, Stockholm, Sweden; 3 School of Health, Care and Social Welfare, Physiotherapy, Mälardalen University, Mälardalen, Västerås, Sweden

**Keywords:** risk factor, young, injury

## Abstract

**Background/aim:**

The injury risk is high in adolescent elite athletes. However, little is known about how the injury risk changes when young talented athletes start studying at a sports high school. The primary aim was therefore to explore the risk of injury when the athlete starts to study at a sports high school. A secondary aim was to identify risk factors for injury.

**Methods:**

A total of 489 athletes (age 15–16 years) were followed for 20 weeks, including 10 weeks before and 10 weeks after the athlete had started to study at a sports high school. Substantial injury was monitored in adolescent elite athletes using the Oslo Sports Trauma Research Centre Questionnaire.

**Results:**

The results showed that the mean difference (md) in injury prevalence was significantly (p=0.001) higher across the 10 weeks after school had started (md 3.6; 95% CI 1.5 to 5.8), compared with the 10 weeks before. Female athletes had significantly (p<0.001) higher injury prevalence (md 6.4%; 95% CI 3.0 to 9.8) across the 10 weeks after school had started, whereas male athletes (md 0.9%; 95% CI −1.8 to 3.6) had not (p=0.530). Three significant (p<0.05) risk factors were identified; previous injury within the past 12 months (OR 3.23), higher training volume (OR 0.97) and lower well-being (OR 0.71).

**Conclusions:**

Our results provide supporting evidence for increased injury risk in female adolescent elite athletes after the athletes had started to study at a sports high school.

**WHAT IS ALREADY KNOWN ON THIS TOPIC**

- The injury risk is high in adolescent elite athletes.

**WHAT THIS STUDY ADDS**

- Female adolescent elite athletes had an overall increased injury risk across the 10 weeks after high school had started, compared with the 10 weeks before. The injury risk was not evident in male adolescent elite athletes.

**HOW THIS STUDY MIGHT AFFECT RESEARCH, PRACTICE OR POLICY**

- Coaches need to be aware of the increased injury risk in female adolescent elite athletes and avoid great changes in load when the athletes are admitted to a sports high school.

## Introduction

Yearly, thousands of young ambitious adolescent athletes decide to study at a national sports high school to improve athletic performance and sport-specific skills while completing a high school degree. Pursuing elite levels of sport is accompanied by greater demands, for example, increased training duration, intensity or competitiveness.^1^ In addition, high levels of exposure to sports in young athletes are likely to increase the risk for sports injuries^2 3^ and negative consequences of early sport specialisation such as sport drop-out rate and unhealthy symptoms (eg, stress, anxiety and burnout) have been highlighted.^4 5^ Still, there is conflicting evidence regarding the influence of sports specialisation on injury risk. In a study by Moseid *et al*,^6^ early sports specialisation was not associated with injury. In addition, unhealthy sleep has been linked to increased injury risk in adolescent athletes.^7–9^ However, less is known about how the injury risk changes once athletes start studying at a sports high school.

Typically, adolescent athletes are between 15 and 16 years of age when they start studying at a sports high school. The education lasts for 3 or 4 years and at the school the athletes can combine elite sports and a high school education. Compared with standard high schools, sports high schools have employed sports coaches who are responsible for the sports education. In Sweden, the long-term goal of the education is to help athletes reach the international elite level in their sport.

Adolescent athletes face injury risk in a period characterised by rapid physical growth and sports activity is often the reason for injury in adolescents.^2 10 11^ The injury incidence is higher during the competition (22.4/1000 hours of competition) compared with training (6.4/1000 hours of training) in adolescent elite athletes.^12–14^ In adolescent elite athletes from different Sport Academy High Schools, the average weekly prevalence of substantial injury has been estimated to 17%.^15^ In elite junior tennis players and handball players, the prevalence of substantial injury has been estimated to 15%.^16 17^ However, there is a lack of prospective injury registration studies in adolescent elite athletes, in contrast to adult elite athletes. This makes it difficult to develop efficient injury prevention programmes due to limited epidemiological data.

By exploring risk factors for injuries in adolescent elite athletes, the athletes with the highest injury risk could be identified. Such findings could be useful when developing efficient injury prevention programmes. Adolescent elite athletes are likely to be exposed to several different risk factors, related to increased training load^1^ and changes in social support and responsibility for the young athlete.^18^ Yet, to the best of our knowledge, no study has been conducted to explore if the school start is associated with increased injury risk. The primary aim was therefore to explore the risk of injury when the athlete starts to study at a sports high school. A secondary aim was to identify risk factors for injury.

## Methods

This study is approved by the Regional Ethical Committee in Sweden and is part of the Karolinska Athlete Screening Injury Prevention (KASIP) study.

### Recruitment process, participants and data collection

Recruitment of athletes was performed in March–May 2019. The heads of all National Federations in Sweden with sports high schools were invited to participate in the KASIP study. This resulted in acceptance from the National Federation of Bandy, Basketball, Canoe, Curling, Football, Gymnastics, Ice-hockey, Orienteering, Sailing, Skiing, Swimming, Tennis and Volleyball.

Approximately 700 adolescent elite athletes (58% boys, age range 15–16), representing 15 different sports, that had applied to start studying at a sports high school were considered eligible and therefore invited. A total of 489 athletes (70%) accepted the invitation, representing sports such as Bandy (n=2), Basketball (n=11), Canoe (n=3), Cross-country skiing (n=51), Curling (n=4), Down-hill skiing (n=21), Football (n=185), Gymnastics (n=19), Ice-hockey (n=74), Orienteering (n=26), Sailing (n=6), Ski cross (n=4), Swimming (n=44), Tennis (n=16) and Volleyball (n=23). The distribution of male and female athletes was similar across all sports, except for Ice-hockey and Volleyball where the majority were male athletes. The included athletes are seen as elite athletes since only athletes with the highest ranking of their age are eligible for the sports high school.

Consent to participate was obtained from the athletes. The Oslo Sports Trauma Research Center (OSTRC) Overuse Injury Questionnaire^19^ was distributed biweekly to the athletes using text messages and was estimated to take 3–5 min to complete. In case of a non-response, a reminder email was sent 4 days later. Athletes were followed 10 weeks before and 10 weeks after the athlete had started studying in a sports high school. The average mean response rate to the questionnaire, across the study period, was 86%. The athletes also fill out an online background questionnaire, during the first week of the study.

### Questionnaire and injury definition

The background questionnaire contained questions about personal data including the history of previous injury, previous illness, following an individual exercise programme, age when deciding one sport as being more important than other sports (referred to as sports specialisation), nutrition and access to medical personnel. A previous injury or illness was defined as an injury or illness sustained within the last 12 months that had affected or completely hindered training for a continuous period of at least 3 weeks. Following an individual exercise programme refers to if the athlete follows an individualised programme, designed by their coach or other personnel associated with sports. This was asked as individualised exercise approaches may be associated with reduced injury risk. Access to medical personnel referred to if the athlete had access to medical personnel if needed.

The biweekly questionnaire contained the validated version of the OSTRC Overuse Injury Questionnaire^19 20^ and measures injury consequences on sports participation, performance, training and pain based on four questions. The OSTRC Overuse Injury Questionnaire has been validated in junior and adult athletes and has been determined to have good face, content and construct validity.^19^ Questions about performed training (hours/week), average training intensity and perceived well-being, as an average mean measure across the two previous weeks, were also added. Training intensity was estimated on a 0–10 scale,^21^ where 0 represents no intensity and 10 is the maximal intensity. Perceived well-being was estimated on a 0–10 scale,^21^ where 0 represents the worst state of well-being and 10 is the best state of well-being. Athletes were categorised as having a substantial injury if they reported problems leading to moderate or severe reductions in training volume, or moderate or severe reduction in performance, or complete inability to participate in sports, based on the responses from the OSTRC Overuse Injury Questionnaire.^19^ Substantial injury was chosen as the outcome as it is a well-defined injury definition, based on OSTRC Overuse Injury Questionnaire.

### Data analysis

Independent sample t-test and χ^2^ test were used to compare differences in background data between sex. The prevalence of substantial injury was estimated at each time point and calculated as a mean value for the period 10 weeks before and 10 weeks after the athlete had started to study at a sports high school. Mean difference (md) of injury prevalence was calculated for all athletes, by sex and sports, and presented with 95% CI. For instance, if the mean injury prevalence was 10% across the 10 weeks before school had started and 15% across the 10 weeks after school had started, the md was estimated to be 5% (15%−10%=5%). This means that the mean injury prevalence was 5% higher across the 10 weeks after school had started. The 95% CI is symmetrical and calculated for the difference between two of the prevalence values. P values, two-sided, were then derived from the 95% CI.

A binomial generalised linear mixed model (GLMM) was used to model the risk of substantial injury (dichotomised outcome) across time (20 weeks) with random intercept by athlete. Possible independent variables were categorical variables (ie, sex, previous injury within the past 12 months, previous illness within the past 12 months, access to medical personnel, following an individual exercise programme, age when specialising in sport) and continuous variables (ie, training volume, training intensity, well-being). Interaction was tested by sex and time and included in the final model if significant (p<0.05). All independent variables associated with the dependent variable at p<0.20, in univariate regression analyses, were included in a backward binomial GLMM. Independent variables were then removed ‘one by one’ based on information criteria such as Akaike information criterion (AIC) and Bayesian information criterion (BIC) values, and the final model was chosen based on these values.

Assumptions of the final model were checked by exploring the normal distribution of random effects and multicollinearity of the included variables. Throughout calculations, the significance level was set to p<0.05. All analyses were conducted using the R statistical system V.3.5.2 (R Foundation for Statistical Computing, Vienna, Austria, 2021).

## Results

Of all athletes (n=489), 38% (n=186) reported previous injury within the past 12 months and 14% (n=69) reported substantial injury at the study start (table 1). There was no difference in background data at baseline between sex, except that a significantly (p=0.017) higher proportion of male athletes (72%, n=192) followed an individual exercise programme, compared with female athletes (62%, n=137).

**Table 1:** Descriptive data by all athletes, female and male athletes

	All athletes (n=489)	Females (n=222)	Males (n=267)	P value*
Access to medical personnel, n (%)	319 (65)	138 (62)	181 (68)	0.193
Age sports specialisation, mean (SD)	12.3 (1.8)	12.3 (1.9)	12.4 (1.8)	0.606
BMI, mean (SD)	21.7 (3.0)	21.7 (3.8)	21.8 (2.3)	0.707
Previous injury,† n (%)	186 (38)	79 (36)	107 (40)	0.309
Previous illness,† n (%)	51 (10)	22 (10)	29 (11)	0.732
Specific exercise programme, n (%)	329 (67)	137 (62)	192 (72)	0.017

*P values based on χ^2^ test and independent t-test for differences between females and males.

†Sustained injury or illness within the last 12 months that has affected or completely hindered training for a continuous period of at least 3 weeks.

BMI, body mass index.

**Figure 1:**
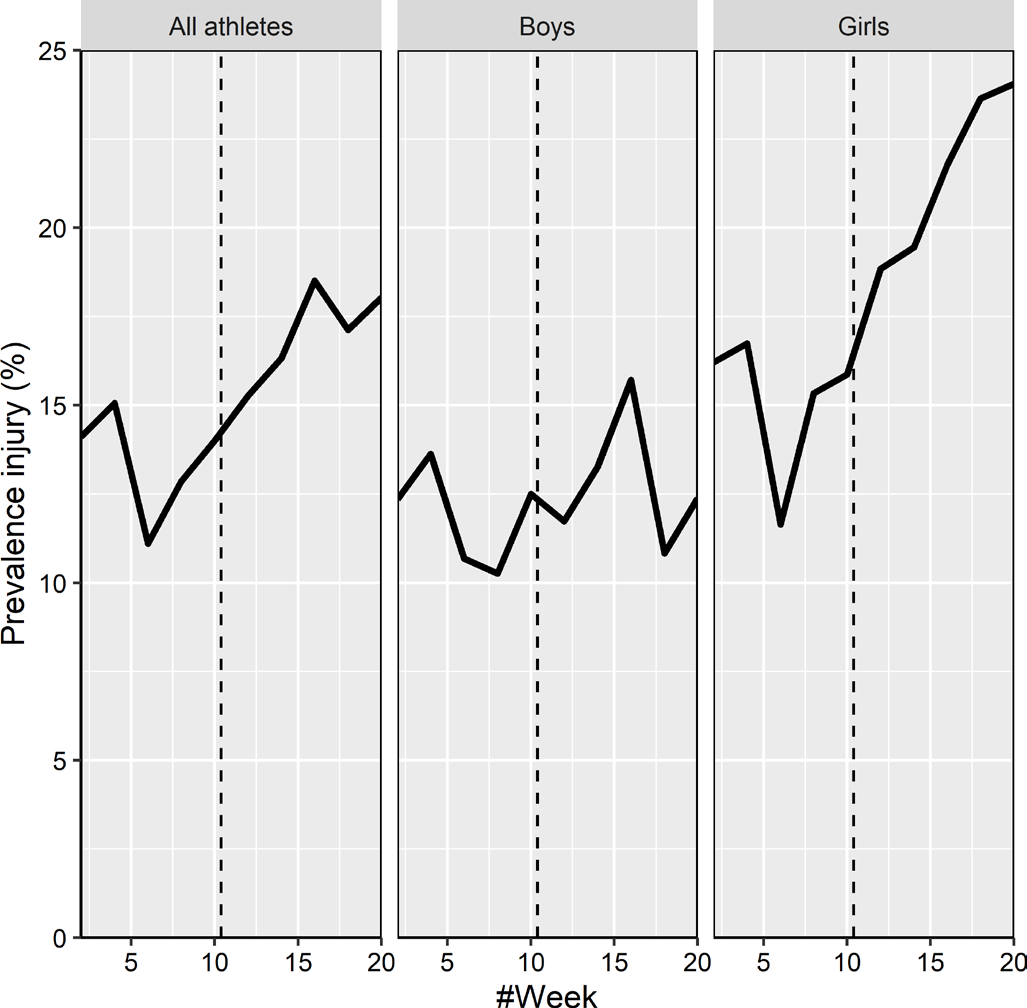
Injury data across time for all athletes, boys and girls with dotted lines illustrating when athletes started studying at a sports high school.

Across the 20 weeks, the mean injury prevalence was 15% (range 20-week period, 11%–19%). The mean injury prevalence was significantly (p=0.001) higher across the 10 weeks after school had started (md 3.6%; 95% CI 1.5 to 5.8), compared with the 10 weeks before (figure 1, table 2). Female athletes had significantly (p<0.001) higher mean injury prevalence (md 6.4%; 95% CI 3.0 to 9.8) across the 10 weeks after school had started, whereas male athletes (md 0.9%; 95% CI −1.8 to 3.6) had not (p=0.530). Injury risk by sports and sex is presented in table 2.

**Table 2:** Difference in injury prevalence across the 10 weeks after high school had started, compared with the 10 weeks before high school had started

Sports	Female/male athletes, n (% females)	Mean difference of injury prevalence (%)*			
		All athletes	P value†	Female	Male
All sports	222/267 (45.4)	3.6 (1.5 to 5.8)	0.001	6.4 (3.0 to 9.8)	0.9 (−1.8 to 3.6)
Basketball	7/4 (63.4)	18.9 (3.6 to 34.2)	0.016	6.2 (−2.2 to 14.5)	53.3 (23.2 to 83.5)
Cross-country skiing	28/23 (54.9)	1.8 (−0.2 to 3.9)	0.082	9.0 (−21.0 to 3.0)	5.6 (−13.7 to 2.4)
Down-hill skiing	12/9 (57.1)	1.1 (−8.5 to 6.4)	0.791	1.5 (−13.6 to 10.6)	0.3 (−6.3 to 6.8)
Football	94/91 (50.8)	3.2 (−0.4 to 6.8)	0.081	6.7 (1.5 to 11.8)	0.7 (−5.6 to 4.3)
Gymnastic	16/3 (84.2)	22.6 (8.9 to 36.3)	0.001	17.6 (2.7 to 32.6)	40.0 (8.0 to 72.0)
Ice-hockey	5/69 (6.8)	3.7 (−0.9 to 8.4)	0.117	9.0 (−21.0 to 3.0)	4.7 (−0.3 to 9.6)
Orienteering	14/12 (53.8)	3.2 (−13.7 to 7.2)	0.558	0.5 (−15.0 to 16.0)	9.2 (−21.3 to 2.9)
Swimming	23/21 (52.3)	5.2 (−0.4 to 10.7)	0.069	7.0 (−2.0 to 16.0)	2.5 (−2.7 to 7.7)
Tennis	7/9 (43.8)	15.2 (−30.7 to 0.4)	0.055	11.0 (−30.3 to 8.4)	20.0 (−42.4 to 2.4)
Volleyball	7/16 (30.4)	5.3 (−4.8 to 15.4)	0.308	9.6 (−13.1 to 32.4)	3.7 (−6.7 to 14.2)
Remaining sports‡	9/10 (47.4)	5.9 (−22.8 to 34.7)	0.699	28.3 (−34.8 to 91.3)	6.9 (−22.7 to 8.8)

Values are presented as mean differences of injury prevalence for all sports, sex and different sports, with 95% CI in parentheses.

*Positive values of injury prevalence should be interpreted as the injury prevalence was increased across the 10 weeks after school had started, compared with the 10 weeks before school had started.

†P values, two-sided, are derived from the 95% CIs.

‡Bandy, Canoe, Curling, Sailing, Ski cross.

**Figure 2:**
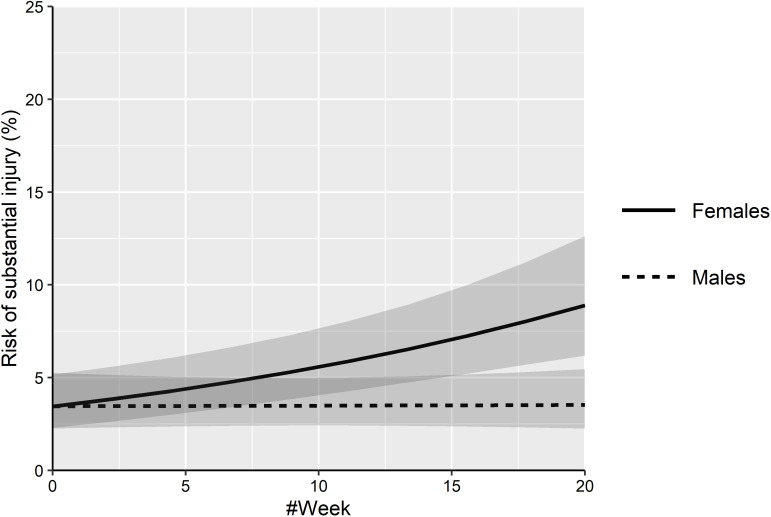
Risk of substantial injury for females and males across time, based on a generalised mixed binomial model, with 95% CIs depicted in grey. Darker grey indicates CIs that overlap.

Univariate analyses identified several significant (p<0.05) risk factors for injury (table 3). Based on a final multivariable binomial GLMM, three risk factors were identified. Having a previous injury within the past 12 months (OR 3.23) and lower well-being (OR 0.71) were significantly (p<0.001) associated with an increased risk of a substantial injury. Higher training volume (OR 0.97) was significantly (p<0.01) associated with reduced risk of substantial injury. In addition, a significant interaction (p=0.003) between sex and time was identified (table 4, figure 2).

**Table 3:** The risk of substantial injury, based on univariate analyses using generalised mixed binomial models

	OR (95% CI)	SE	P value
Access to medical personnel	0.89 (0.67 to 1.22)	0.23	0.369
Age sports specialisation	0.94 (0.84 to 1.05)	0.06	0.253
BMI	1.10 (1.03 to 1.17)	0.03	0.003
Perceived training intensity	0.89 (0.83 to 0.96)	0.04	0.004
Previous injury			
Have had a previous injury*	3.36 (2.23 to 5.05)	0.21	<0.001
Previous illness			
Have had a previous illness*	2.48 (1.32 to 4.67)	0.32	0.005
Sex (female athlete)	2.08 (1.37 to 3.16)	0.21	<0.001
Specific exercise programme	0.84 (0.54 to 1.32)	0.23	0.457
Training volume	0.96 (0.94 to 0.97)	0.01	<0.001
Well-being	0.69 (0.64 to 0.73)	0.03	<0.001

*Sustained injury or illness within the last 12 months that has affected or completely hindered training for a continuous period of at least 3 weeks.

BMI, body mass index.

**Table 4:** Generalised mixed binomial model for substantial injury

Model*	OR (95% CI)	SE	P value
Time	1.12 (1.06 to 1.18)	0.03	<0.001
Sex			
Female athlete	1.12 (0.64 to 1.95)	0.28	0.694
Interaction time*sex	0.90 (0.83 to 0.96)	0.04	0.003
Previous injury			
Have had a previous injury†	3.23 (2.19 to 4.79)	0.2	<0.001
Training volume	0.97 (0.96 to 0.99)	0.01	<0.001
Well-being	0.71 (0.67 to 0.76)	0.03	<0.001

*Intercept 2.66

†Sustained injury within the last 12 months that has affected or completely hindered training for a continuous period of at least 3 weeks.

## Discussion

The main finding was that female adolescent elite athletes had an overall increased injury risk across the 10 weeks after school had started, compared with the 10 weeks before. The injury risk was not evident in male adolescent elite athletes, although great differences in injury risk occurred across different sports. Previous injury, lower well-being and higher training load were identified as risk factors for injury.

To the best of our knowledge, the novel finding of increased injury risk in female adolescent elite athletes across the 10 weeks after high school had started has not been demonstrated before. When adolescent elite athletes enter a sports high school, a cascade of changes occurs during a relatively short period,^18^ such as changes in social support and responsibility. At sports high schools, the athletes also continue their trajectory into elite sports, accompanied by greater demands and increased competitiveness.^2^ It is therefore not remarkable that athletes face an increased injury risk. We believe the increased injury risk of 3.6% is clinically relevant as it refers to a difference in the prevalence of substantial injury. However, this estimate varied greatly across sports.

Previous research has shown that female athletes have a different injury profile than male athletes. For instance, female adolescent athletes have an increased risk for overuse injuries in general,^22^ stress fractures,^23^ concussions^24^ and ankle and knee injuries,^25^ compared with adolescent males. The incidence of anterior cruciate ligament injuries is higher in adolescent female athletes compared with male athletes.^26–28^ In addition, menstrual disturbances are common among adolescent elite athletes,^29^ which might also be related to injury risk. Even if there was no overall increased injury risk in male athletes, there was wide variation in injury risk across sports.

A previous injury was the strongest risk factor for injury identified in this study. This has been suggested to be related to inadequate rehabilitation or to a specific injury risk behaviour or trait associated with the previously injured athlete.^30 31^ Interestingly, 14% of the athletes were injured at the study start, 10 weeks before school start. This suggests that a considerable part of injury problems is not caused or related to the school start. It also suggests that some athletes are likely to bring their current injury problem to the sports high school, highlighting the importance of providing adequate rehabilitation close to school start. A decrease in well-being and increased training volume were also associated with increased injury risk. Previous research has shown that negative life-event stress^32 33^ or daily hassles,^34 35^ likely associated with well-being, have been found to influence injury risk. Even if exposure to sports and competitiveness is likely increased at sports high school, our findings highlight that training load should not be considered as a risk factor for injury in this context. However, it is likely that training load acts as a proxy for sports participation, as training load varies across sports. Therefore, some of the association between training load and injury risk is probably confounded by the specific sport the athlete participates in. In this study, we could not control for type of sports as several sports had few participants. Even if training volume was found to be protective against injury, it should be acknowledged that the upper limit of the CI was close to 1, suggesting it had a small effect on injury risk and should be interpreted with caution.

We believe our findings are important for coaches and medical personnel who meet and work with adolescent elite athletes. They should be aware that (1) a considerable part of adolescent elite athletes is currently or have recently been injured, (2) the injury risk is increased in female athletes and (3) previous injury is a major risk factor. It is also important that adolescent elite athletes with a previous injury are identified as they have a high risk for a new injury or recurrent injury. Future studies should explore coaches’ and medical personnels’ experiences and thoughts about injury prevention for adolescent elite athletes who are admitted to sports high schools.

The strength of this study is the prospective nature, following a high number of adolescent elite athletes aged 15–16 from 15 different sports before they are admitted to a sports high school. To attend these schools, all athletes have to compete at the highest national level for their age group, making a homogeneous group of adolescent elite athletes. A reliable, valid questionnaire previously used in sports surveillance was also used, as well as a modern definition of injury with an adequate response rate across the study period. The findings of this study should also be viewed in light of potential limitations. We only followed the athletes for 20 weeks. This was decided on the basis that we only could include athletes approximately 10 weeks before school start and we wanted to have an equal period before and after the athletes started to study. Therefore, we have limited data (eg, training load, competition, injury severity) prior to the study start, even if we collected data on the occurrence of previous injury. Training load was self-reported, and consequently, both overestimation and underestimation need to be considered. We also lacked data on physiological maturity, which has been found to influence injury risk in junior football players.^36^ The injury risk in specific sports should also be interpreted with caution, as several sports had a small sample of athletes. In addition, the included athletes have different season schedules (eg, base training, pre-season, competitive seasons), which may have led to different injury risks at the study start related to competition exposure.

## Conclusions

Our results provide supporting evidence that female adolescent elite athletes have an increased risk of injury across the 10 weeks after high school had started, compared with the 10 weeks before. Male adolescent elite athletes did not have an overall increased injury risk. In addition, athletes with a previous injury, low well-being and a high training load had the highest risk for injury.

## Data Availability

Data are available upon reasonable request. The data that support the findings of this study are available on reasonable request from the corresponding author.
